# Quantification of IFNγ- and IL17-producing cells after stimulation with citrullinated proteins in healthy subjects and RA patients

**DOI:** 10.1007/s00296-012-2470-9

**Published:** 2012-07-24

**Authors:** Katleen Van Steendam, Marlies De Ceuleneer, Kelly Tilleman, Dirk Elewaut, Filip De Keyser, Dieter Deforce

**Affiliations:** 1 Laboratory for Pharmaceutical Biotechnology, Ghent University, Harelbekestraat 72, 9000 Ghent, Belgium; 2 Department of Rheumatology, Ghent University Hospital, De Pintelaan 185, 9000 Ghent, Belgium

**Keywords:** Citrullination, Rheumatoid arthritis, ELISpot, T cells

## Abstract

Antibodies against citrullinated proteins are highly specific for rheumatoid arthritis (RA) and are currently used as a diagnostic marker. In this study, we wanted to quantify the numbers of T cells that react to a wide range of citrullinated proteins in a wide range of HLA-DR subtypes in order to investigate whether citrullination might create T-cell neo-epitopes and could initiate a universal T-cell response. Therefore, PBMCs from healthy volunteers and RA patients were stimulated with a citrullinated and non-citrullinated cell extract on IFNγ-ELISpot. We found a significantly higher number of IFNγ-secreting cells after stimulation with citrullinated proteins compared to non-citrullinated proteins in RA patients (1:14,441 cells vs. 1:32,880 cells) as well as in healthy subjects (1:6,261 reactive cells compared to 1:16,212 cells). Additionally, a higher number of IL17-secreting cells were found after stimulation with citrullinated proteins compared to their non-citrullinated counterparts. Our data indicate that citrulline-dependent T-cell response is not restricted to RA patients but that citrullination as such gives rise to a universal break in tolerance.

Reactivity against citrullinated proteins is an important part of the rheumatoid arthritis (RA) pathology, and the presence of antibodies against citrullinated proteins (ACPA) predisposes for a more severe disease course [1]. Since ACPA are class-switched antibodies, a citrulline-specific T cell is likely involved in their production [2].

Several papers investigated the presence of citrulline reactive T cells. Recently, Snir et al. detected citrullinated vimentin aa 59-78-reactive T cells in both RA patients and healthy volunteers by means of HLA-DRB1*0401 tetramers. Moreover, similar percentages of DR0401-positive cit-vimentin aa 59-78 cells were observed in RA patients and controls samples [3]. James et al. tested several citrullinated peptides for their binding capacity to HLA-DRB1*1001 [4]. The choice of peptides for these assays was based on prediction models for the binding to HLA-DRB1*1001, similar to Snir et al. [3] where choice was based on potential binding to HLA-DRB1*0401. However, the authors remarked that peptides from prediction models might not be naturally processed and presented and that it is likely that other epitopes are present among the peptides that were not studied [4].

Another recent study by Feitsma et al. investigated reactivity against two peptides from citrullinated vimentin and proved that these could be naturally processed [5]. They found that 3 of the 10 RA patients showed higher IFNγ response on citrullinated peptides compared to their non-citrullinated counterpart, while no significant difference was found in healthy controls. The main disadvantage of this study is the use of only 2 vimentin peptides and the selection of HLA-DRB1*04-positive patients and controls, further restricting the reactivity under investigation.

Therefore, the need emerged for an experiment that used a broad range of citrullinated proteins. In fact, it is possible that while the epitopes tested in the above-mentioned papers do not have a differential amount of reactive T cells, other epitopes might. To circumvent the problem of T cell assays that use a limited amount of peptides, we chose to use whole citrullinated proteins for the stimulation of PBMCs, which also indicates the involvement of antigen-presenting cells and therefore mimics the situation in the body regarding antigen processing more accurately. Additionally, to the authors’ knowledge, the number of reactive T cells that react to citrullinated epitopes has never been quantified before.

An additional downside of the use of peptides and prediction models is their restriction to certain HLA types. The above-mentioned papers restrict their analyses to HLA-DRB1*1001 [4] or HLA-DRB1*0401 [3, 5], but other HLA types are associated with ACPA such as HLA-DRB1*0101 and 0404 (7). Therefore, patients with a broad range of HLA subtypes should be used in an experiment on the T cell reactivity to citrullinated proteins (Table 1).

**Table 1:** Patient information and clinical data: the rheumatoid factor (U/ml) and CCP (U/ml) titre and HLA-DRB1 status of the different RA patients and healthy volunteers used throughout the study

Patient* n*°	Gender	Age	RF (U/ml)	CCP (U/ml)	HLA DRB1
Healthy controls					
1	F	22	ND	ND	01/08
2	F	45	<7.9	0.3	01/04
3	F	32	<7.9	0.4	04/13
4	M	37	<7.9	0.4	13/15
5	M	32	<7.9	0.4	01/07
6	F	45	<7.9	1.4	04/11
7	F	45	<10.6	0.4	11/11
8	F	24	ND	ND	07/14
9	F	28	ND	ND	03/09
10	M	26	ND	ND	ND
11	M	63	<10.6	1.1	11/13
12	F	26	ND	ND	ND
13	F	30	ND	ND	ND
14	F	61	<10.6	1	04/15
15	F	62	<10.6	1.6	07/15
16	M	60	<10.6	0.6	01/04
17	M	61	<10.6	1.6	11/15
18	F	60	<10.6	0.2	13/15
19	F	83	<10.6	1.2	01/13
RA CCP+					
1	F	68	105	75	04/15
2	M	63	13.3	55	01/16
3	M	67	100	199	03/15
4	F	63	66.8	83	01/04
5	F	72	30.8	75	04/04
6	F	81	<10.6	>Max	01/03
7	F	65	156	611	04/04
RA CCP−					
1	M	67	<7.9	0.4	03/07
2	M	73	<7.9	0.6	01/15
3	M	67	<7.9	0.5	01/07
4	F	68	<7.9	1.1	04/04
5	M	62	<7.9	0.5	13/16
6	F	46	<7.9	0	07/11
7	F	60	40.4	0.2	ND

*ND* not determined

This study is the first to quantify the number of T cells reactive to a broad range of non-citrullinated and in vitro citrullinated proteins from a human cell lysate in patients and healthy controls with different HLA-DRB1 subtypes.

In order to accurately count the low numbers of citrulline reactive T cells, we used IFNγ-ELISpot (Mabtech) (20 μg of non-citrullinated or citrullinated proteins per 500,000 PBMCs). In both RA patients and healthy controls, a statistically significant higher number of reactive PBMCs was found after stimulation with citrullinated proteins compared to their non-citrullinated counterpart (Fig. 1 upper panel). Our healthy subjects (*n* = 19) had on average 1:6,261 PBMCs reactive to citrullinated proteins, while 1:16,212 PBMCs on average was reactive against the non-citrullinated proteins. In the RA group (*n* = 10), on the other hand, an average of 1:14,441 cells and 1:32,880 cells was found to be reactive upon stimulation with citrullinated and non-citrullinated proteins, respectively. The number of spot-forming counts after stimulation with citrullinated proteins was significantly increased compared to stimulation with their non-citrullinated counterpart in both healthy volunteers and RA patients.

**Fig. 1:**
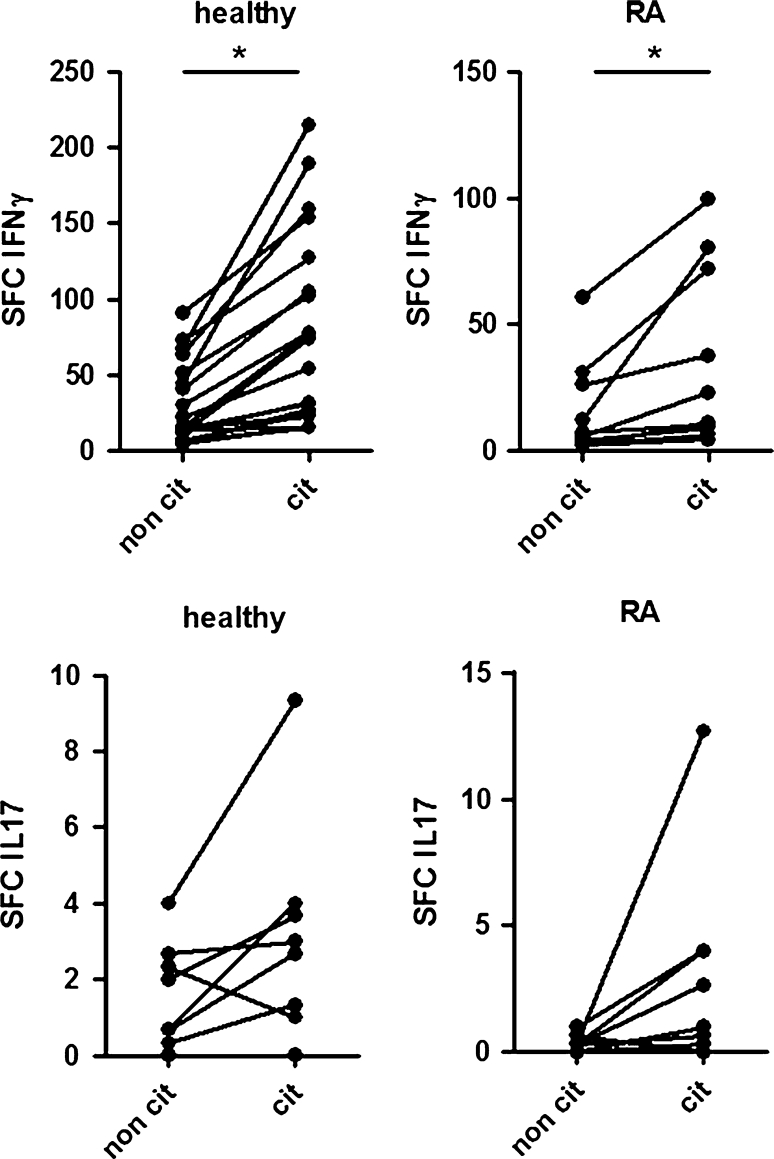
IFNγ and IL17-reactivity of PBMCs from RA patients and healthy controls after stimulation with non-citrullinated (non cit) and citrullinated (cit) proteins. *Top panel* IFNγ spot-forming counts of 500,000 PBMCs of healthy volunteers (*n* = 19) and RA patients (*n* = 10). A significantly higher amount of spots could be counted after stimulation with citrullinated proteins for both groups. *Bottom panel* IL17 spot-forming counts of 500,000 PBMCs of healthy volunteers (*n* = 8) and RA patients (*n* = 9). No statistical analysis was performed due to low spot counts, but a trend towards higher frequency of IL17-secreting cells after stimulation with citrullinated proteins was detected

Besides making the distinction between RA patients and healthy subjects, it might be relevant to differentiate between ACPA+ and ACPA−. Our data reveal no significant difference between ACPA+ and ACPA−: ACPA+ (*n* = 5) and ACPA− (*n* = 5) patients showed a respective average frequency of 1:16,484 and 1:12,397 reactive cells after stimulation with citrullinated proteins and 1:34,247 and 1:31,513 after stimulation with their non-citrullinated counterparts.

The observed T cell reactivity nearly disappeared (drop of >96 % in reactivity for stimulation with citrullinated as well as non-citrullinated proteins) when PBMCs were depleted for HLA-DR, indicating that APCs are crucial for the anticitrulline response. However, as mentioned before, several subtypes of this HLA can be involved in this response.

Additionally, we analysed spot-forming counts for synovial fluid mononuclear cells from 4 RA patients. Although the sampled population was small, we could see a greater IFNγ response after stimulation with citrullinated proteins compared to non-citrullinated proteins (1:3,846 and 1:5,249, respectively). Overall, there were more reactive T cells in synovial fluid than in peripheral blood of RA patients, which confirmed the findings made by Rönnelid et al. [6].

Besides IFNγ production, we also analysed IL17 production of healthy and RA PBMCs by means of IL17-ELISpot. The number of IL17-secreting cells after stimulation with citrullinated proteins and non-citrullinated proteins was rather low. Healthy subjects (*n* = 8) had on average 1:160,000 reactive T cells after stimulation with citrullinated proteins and 1:315,706 after stimulation with non-citrullinated proteins. RA patients (*n* = 9) had on average 1:177,585 reactive cells after stimulation with citrullinated proteins and an average of less than 1 reactive cell per 500,000 PBMCs after stimulation with non-citrullinated proteins. No statistical analysis was performed, due to the low spot count. However, a trend towards a higher frequency of IL17-secreting cells after stimulation with citrullinated proteins was detected (Fig. 1, lower panel). These data are in agreement with the results obtained by Snir et al. [3], who could also detect a trend towards a higher frequency of IL17-secreting cells after stimulation with a citrullinated vimentin peptide loaded on MHC tetramer, but no significant difference. Additionally, it should be noted that our analysis used a broader range of proteins on a diverse HLA background, while Snir et al. only used one peptide on a certain HLA subtype as stimulant (citrullinated vimentin aa 59-78 on HLA-DRB1*0401) [3].

In conclusion, several groups have recently reported T cell reactivity against citrullinated synthetic peptides or recombinant proteins in healthy individuals [3, 7] and RA patients [5, 8]. All these studies restricted their experiments to stimulation of lymphocytes of a certain HLA-DRB1 subtype with a limited amount of citrullinated synthetic peptides or recombinant proteins and will therefore only detect reactivity in susceptible individuals with the right HLA type and for the specifically studied peptide(s) or proteins. Our study is the first to quantify and characterize the whole citrulline reactive T cell pool independent of HLA-DR background. We could not detect any differences in number of citrulline reactive T cells when comparing healthy and RA PBMC. This implies that the presence of T cells with citrulline specificity alone is not sufficient for RA pathology and confirms the existing presumption that citrulline reactive T cells escape thymic selection [2].

We did, however, detect a significant difference in spot-forming count between IFNγ production after stimulation of PBMC with citrullinated and non-citrullinated proteins for both healthy volunteers and RA patients, likely because we did not confine our study to a single citrullinated peptide. This confirms the presence of autoreactive T cells against citrullinated epitopes in healthy individuals and suggests that citrullination of proteins creates neo-epitopes that escape thymic selection during T-cell maturation.
